# Altering cell death pathways as an approach to cure HIV infection

**DOI:** 10.1038/cddis.2013.248

**Published:** 2013-07-11

**Authors:** A D Badley, A Sainski, F Wightman, S R Lewin

**Affiliations:** 1 Division of Infectious Diseases, Department of Medicine, Mayo Clinic, Rochester, MN, USA; 2 Department of Molecular Medicine, Mayo Clinic, Rochester, MN, USA; 3 Department of Molecular Pharmacology, Mayo Clinic, Rochester, MN, USA; 4 Department of Infectious Diseases, Alfred Hospital and Monash University, Melbourne, Victoria, Australia; 5 Centre for Biomedical Research, Burnet Institute, Melbourne, Victoria, Australia

**Keywords:** apoptosis, latency, HIV cure, Casp8p41, Bcl2

## Abstract

Recent cases of successful control of human immunodeficiency virus (HIV) by bone marrow transplant in combination with suppressive antiretroviral therapy (ART) and very early initiation of ART have provided proof of concept that HIV infection might now be cured. Current efforts focusing on gene therapy, boosting HIV-specific immunity, reducing inflammation and activation of latency have all been the subject of recent excellent reviews. We now propose an additional avenue of research towards a cure for HIV: targeting HIV apoptosis regulatory pathways. The central enigma of HIV disease is that HIV infection kills most of the CD4 T cells that it infects, but those cells that are spared subsequently become a latent reservoir for HIV against which current medications are ineffective. We propose that if strategies could be devised which would favor the death of all cells which HIV infects, or if all latently infected cells that release HIV would succumb to viral-induced cytotoxicity, then these approaches combined with effective ART to prevent spreading infection, would together result in a cure for HIV. This premise is supported by observations in other viral systems where the relationship between productive infection, apoptosis resistance, and the development of latency or persistence has been established. Therefore we propose that research focused at understanding the mechanisms by which HIV induces apoptosis of infected cells, and ways that some cells escape the pro-apoptotic effects of productive HIV infection are critical to devising novel and rational approaches to cure HIV infection.

## Facts

- Human immunodeficiency virus (HIV) has been cured in one and possibly more patients.
- Efforts are underway to recapitulate a cure for HIV in a generalizable way.
- Cells containing latent HIV that are induced to reactivate virus do not die due to viral replication.
- Chronically HIV-infected cells are resistant to apoptosis.
- Understanding the regulation of apoptosis during HIV infection and latency may be the key to develop a cure for HIV.

## Open Questions

- Why do all cells that are HIV infected not die as a consequence of productive HIV replication?
- Why do latently HIV-infected cells that are induced to reactivate virus not die as a result of productive HIV replication?
- Can therapeutic strategies be designed that will both reactivate HIV from latency and induce the death of cells that replicate HIV?

Timothy Ray Brown, ‘the Berlin Patient,' is now >6 years post bone marrow transplant (BMT) from a donor with the Δ32 mutation in CCR5, and he has no detectable HIV in his blood or tissues while off combination antiretroviral therapy (cART).^1, 2, 3^ His story has fostered hopes that HIV might be cured, within the foreseeable future. In this article, we will review the recent advances in our understanding of HIV latency, review the approaches that are being tested as a means to cure HIV and discuss the challenges associated with targeting latently HIV-infected CD4 T cells for death, with a focus on ways to alter apoptosis regulation.

## Relationship Between Viral Infection, Apoptosis and Viral Persistence

Broadly speaking, viruses that cause human disease cause either acute self-limited infections or chronic persistent infections, each with differing effects on host cell apoptosis and viral persistence.

Acute self-limited viral infections include influenza, Ebola, Hantavirus and dengue, among others. In the best studied of these, influenza virus, hemaglutinin expressed on the surface of an infectious virion binds sialic acid sugars on the surfaces of epithelial cells, typically in the nose, throat, and lungs of the susceptible host.^4, 5^ Following viral replication, progeny virions bud are released from the infected cell and the cell undergoes apoptosis.^6^ Apoptosis is associated with an interferon response and decreasing the magnitude of this response increases cell survival following challenge with influenza.^7^ The influenza nonstructural (NS) protein-1, also induces caspase-dependent apoptosis,^8^ but to variable degrees according to sequence differences in influenza subtypes,^9^ likely involving interactions with microtubules.^10^ As influenza is an acute self-limited infection there are no mechanisms that allow for influenza persistence, and there are no influenza-associated pathways that inhibit apoptosis.

Chronic persistent or chronic latent infections occur with the herpes virus family (Epstein Barr Virus (EBV), cytomegalovirus (CMV) and herpes simplex virus (HSV)), HIV, Hepatitis B and C, and human papilloma virus (HPV), among others. What distinguishes these infections is their ability to persist long after the initial symptoms of infection have resolved and that each virus has evolved strategies to evade apoptosis. The various strategies used by these viruses to evade cell death have been extensively reviewed elsewhere.^11^

A causal relationship between evasion of apoptosis and the establishment of latency has been demonstrated in the case of EBV. EBV encodes two viral homologs of the cellular anti-apoptosis protein Bcl2, called BALF1 and BHRF1. EBV mutant viruses missing both BALF1 and BHRF1 resulted in robust viral replication but chronic persistent infection as measured by lymphoblastic transformation did not occur.^12^ Thus in the case of EBV, chronicity and transformation depends upon the presence of apoptosis inhibitors, and so opens the possibility that apoptosis inhibition is required for latency in other viral systems.

HIV is a virus that establishes latency, and has developed multiple strategies to inhibit apoptosis of infected cells under a variety of circumstances. We have previously reviewed ways that HIV infection and individual HIV proteins inhibit apoptosis.^13^ Although no HIV proteins are in themselves apoptosis inhibitors, the expression of select HIV proteins, such as Vpr, Nef and Tat, alter the transcriptional profile of some cell types to produce more endogenous apoptosis inhibitory proteins. For example, *in vitro*, Tat induces cellular FLICE inhibitory protein (cFLIP) expression in T cells^14^ including primary T cells and thereby confers resistance to apoptosis.^15^ Vpr increases Bcl2 and decreases Bax in the Jurkat T cell line,^16^ and there is increased X-linked inhibitor of apoptosis (XIAP) expression in latently infected cell line models.^17^ Both Tat-treated monocytes^18^ and primary CD4 T cells from HIV-infected patients^19^ develop tumor necrosis factor (TNF)-related apoptosis inhibitory protein (TRAIL) resistance, potentially through the production of a novel TRAIL splice variant (TRAILshort), which preferentially binds TRAIL receptor (R)2 and prevents pro-apoptotic TRAIL from signaling.^20^ TRAILshort can be found in the plasma and cells of HIV-infected patients.^20^ Another possible mechanism is Tat-mediated upregulation of a variety of nuclear factor kappa-B (NF*κ*B)-dependent apoptosis inhibitors, including Bcl2, cFLIP, XIAP and CIAP.^21^

## HIV Latency in CD4+ T-cells

One of the major barriers to HIV cure is the establishment of latency in resting CD4+ T cells. This may arise from cells that are productively infected with HIV and revert to a resting memory T-cell phenotype with integrated pro-virus (post-activation latency) or via direct infection of resting CD4+ T cells (pre-activation latency). The relative contribution of each pathway *in vivo* is unknown. Once latency is established, latently infected resting memory T cells have a prolonged half-life estimated to be 44 months (reviewed in Finzi *et al.*^22^ and Pierson *et al.*^23^). Latently infected cells are detected at increased frequency in tissue, such as lymphoid tissue and the gastrointestinal (GI) tract.^24^ Virus can also persist in other long-lived cells such as infected macrophages,^25^ naïve T cells,^26^ follicular dendritic cells^27^ and the cells in the central nervous system (CNS) including astrocytes^28^ and microglia.^29^ The relative contribution of these non-memory T-cell reservoirs is less clear.

The molecular basis of latency—defined as HIV DNA integrated in the host genome but remaining transcriptionally inactive—is complex and may involve multiple mechanisms contributing to transcriptional repression simultaneously. This topic has been extensively reviewed elsewhere^30^ and include the following mechanisms: (i) insufficient levels of host transcription factor expression, and/or expression of transcription factor complexes with negative regulatory activity;^31^ (ii) epigenetic silencing and chromatin remodeling that prevent access of transcription factors to transcription initiation sites within the HIV long terminal repeat (LTR);^32^ (iii) differences in the efficiency of HIV transcription between different insertion sites and different insertion orientations;^33^ and (iv) the absence of the transactivator of transcription encoded by HIV, Tat.

## Models of Latency

There are multiple models of HIV latency, which have been used to understand both the establishment and maintenance of latency and to identify molecules that can activate virus production from latently infected cells.^34^ Currently available models include latently infected cell lines, latently infected primary CD4+ T cells and resting CD4+ T cells from patients on suppressive cART (generally defined as plasma HIV RNA <50 copies/ml).

### Latently infected cell lines

The most commonly used latently infected cell lines include ACH2,^35^ U1,^36^ J-lat clones^37^ and J89.^38^ Latently infected cell lines have several limitations. First, they represent clonal populations in which a single integration site is present,^36, 37^ whereas latently infected CD4+ T cells from patients have integration sites randomly distributed throughout the host genome. Second, cell lines are rapidly dividing cells, while latently infected CD4+ T cells found *in vivo* are resting.^39^ This impacts reactivation strategies such as histone deacetylase inhibitor (HDACi), which are 10-fold more active in transformed cells compared with non-transformed cells.^40^ Finally, in latently infected cell lines, integration usually occurs at sites of heterochromatin^37^ while latently infected primary cells CD4+ T HIV integrates into sites of active gene expression.^41^

### Latently infected primary CD4+ T cells

Several primary CD4+ T-cell models of latency exist where activated cells are infected and subsequently allowed to return to a quiescent latently infected state.^42^ One model has used naïve CD4+ T cells that are polarized and then infected with a single round virus (which is envelope deficient). Another uses naïve CD4+ T cells co-cultured with antigen-presenting cells and infected with a wild-type HIV (capable of multiple rounds of infection)^42^ or stimulated with anti-CD3/CD28 before infection. These models are technically demanding as they require a long time in culture ranging from 21 days^43^ to >60 days.^42^ Other models have used direct infection of resting CD4+ T cells either via spinoculation^44^ or in *ex vivo* tonsil tissue blocks or following incubation with chemokines such as CCL19 or CCL21 (ligands for CCR7), which allows for efficient viral nuclear localization and integration without activation of the cell.^45, 46^ Finally, CD4+ T cells can also be transduced with Bcl2 to allow for long-term culture, infected with HIV and permitted to return to a resting state.^47^ The frequency of latently infected cells in these models ranges from 0.1 to 1.0%^42, 46, 47^ to as high as 20–30%.

### Resting CD4+ T cells from HIV-infected patients on cART

The gold standard model of latently infected cells is resting CD4+ T cells from HIV-infected patients on suppressive cART.^48^ The frequency of latently infected cells can be quantified by activation with a mitogen or anti-CD3/CD28 and then co-culturing with uninfected cells to amplify viral production (also called limiting dilution micro-coculture or infectious units per million (IUPM) cells). While this represents the most accurate assessment of latently infected cells *ex vivo*, this technique is time consuming; requires the collection of large volumes of blood usually via leukapheresis from patients; and is only semi-quantitative.^48^ Recent evidence also suggests that IUPM may underestimate the true number of latently infected cells that carry infectious virus and may only represent 10–30% of ‘activatable' virus.^49^

## Viral Latency and the Role of Apoptosis Pathways

If HIV killed all of the cells that it infects, then logically, HIV persistence would not occur. If persistence is the goal, why then does HIV kill any cells that it infects? Simply, the machinery that is activated during apoptosis (caspase activation) also acts through the BCL10/MALT1/CARMA pathway to activate NF*κ*B, which is the most potent activator of HIV transcription, via the HIV LTR.^35^ Evidence supporting this includes: (i) HIV replication is increased in T leukemia cells and peripheral blood mononuclear cells treated with the pan-caspase inhibitor z-VAD-fmk;^50^ (ii) HIV replication in immortalized T-cell lines induced to express the pro-apoptotic proteins FasL, Fas-associated death domain (FADD) protein and p53; (iii) HIV replication is decreased in cells overexpressing the anti-apoptotic proteins Bcl2, FLIP^51^ or with knockdown of the pro-apoptotic proteins Bax or FADD;^52^ and (iv) expression of Casp8p41 (a unique cleavage fragment of procaspase 8 generated by HIV protease) in infected cells directly activates NF*κ*B-dependent HIV LTR transcription.^53^ Given this, one would predict that in settings where the goal of HIV infection is to establish a latent reservoir, it would not be in the interest of HIV to induce cell death.

## Apoptosis Susceptibility in Memory T cells *in Vivo*

Resting memory T cells are metabolically inactive; function to archive historical immune responses; and therefore, need to be long lived and resist stimuli which under normal circumstances favor T-cell death. To understand apoptosis resistance of latently HIV-infected T cells, it is important to first understand the mechanisms of apoptosis resistance in memory CD4 T cells.

T-cell activation and reversion to memory has been the subject of a recent excellent review.^54^ CD4 T cells undergo activation and proliferation after exposure to a neoantigen that is recognized by the T-cell receptor (TCR) and presented in the context of appropriate major histocompatibility complex (MHC) Class II and co-stimulatory help. Resting CD4 T cells that express CCR7 and CD62L are relatively metabolically inactive and apoptosis resistant before they encounter antigen. However, after antigen exposure with appropriate co-stimulation, T cells proliferate, produce interleukin-2, upregulate Fas and other pro-apoptotic molecules, and change from an apoptosis-resistant state to a post-activation apoptosis-prone state. Forty eight hours after antigen exposure, the CD4 T cell is maximally activated and most sensitive to apoptosis. Thus, in settings such as prolonged TCR engagement, absence of co-stimulatory molecules, or withdrawal of cytokines required for survival, these cells undergo activation-induced cell death (AICD).

The principal molecular mediators of AICD are Fas and Fas ligand, but the susceptibility of the cell to undergo Fas-mediated death is governed by the cumulative expression of apoptosis regulatory proteins, including Bcl2 family members, Inhibitor of Apoptosis proteins (IAPs) and cFLIP. Because productive HIV infection pervasively alters the expression of apoptosis regulatory molecules, the fraction of cells that undergo AICD in an HIV-infected patient is greater than that of an HIV-negative patient and this contributes to the depletion of uninfected CD4 T cells.^55^

CD4 T-cell memory occurs when antigen concentrations become low but co-stimulation with CD28 and/or interleukin-2 persists. In such settings, anti-apoptotic molecules such as Bcl2 or cIAPs are upregulated to prevent AICD. This process is coincident with the downregulation of CD45RA and low-level expression of CD45RO. There are two types of CD4 memory T cells: central memory T cells (TCM), which express lymph node-homing molecules CCR7 and CD62L;^56^ and effector memory T cells (TEM), which express receptors such as CCR5.^57^ HIV preferentially infects HIV-specific activated memory CD4+ T cells, as opposed to memory cells of other specificities.^58^ Alternately HIV may infect activated cells, which then adopt a central memory phenotype. We have recently shown that stromal-derived factor (SDF-1*α*) treatment of activated CD4+ T cells resulted in degradation of Bim, resulting in apoptosis resistance and cells adopting a memory phenotype.^59^ Thus, HIV-infected CD4+ T cells with a memory phenotype, including latently infected cells, are apoptosis resistant, making these cells particularly difficult to eradicate.

## Approaches to Cure HIV

Recent advances in our understanding of the biology of HIV have now allowed for proposals to recapitulate either a sterilizing cure (complete elimination of all HIV-infected cells from an individual) or functional cure (long-term control of HIV replication with HIV RNA <50 copies/ml in the absence of cART).

Very early initiation of cART has been associated with a reduced number of latently infected cells and recently there have been reports of successful control of virus replication in individuals who have initiated treatment early and stopped cART.^60^ The frequency of ‘post-treatment control' is extremely rare—estimated at <2% of all patients who initiate ART during acute infection.^61^ Very early initiation of cART following delivery of a baby to an HIV-infected mother not on ART in Mississippi was also recently reported to result in a functional cure.^62^

Three classes of approaches have been proposed as curative strategies for HIV: reactivation approaches, gene therapy-based approaches, and immune-based therapies (Table 1).

### HIV Reactivation

The underlying premise of this approach is that HIV reactivation from a latently infected CD4 T cell will lead to cytotoxicity which, it is hoped, will cause all infected cells to die. A wide variety of approaches have been proposed to reactivate HIV (Table 2). Great interest has focused on epigenetic silencing mechanisms such as histone acetylation, and ways to reverse these processes.

A small proof of concept study of a single dose of the HDACi, vorinostat in HIV-infected patients on suppressive cART resulted in an increase in both histone acetylation and cell-associated HIV RNA in resting memory CD4+ T cells.^63^ We recently completed a multidose study of 14 days of daily vorinostat in HIV-infected patients (*n*=20) and demonstrated an increase in cell-associated HIV RNA in 90% of participants, although disappointingly we observed no decline in HIV DNA.^64^ A similar study is being performed in Denmark with the potent HDACi panobinostat [clinicaltrials.gov] and a single dose study of the HDACi rhomedepsin is underway. Disulfiram, which acts via a totally different mechanism, most likely via depletion of the phosphatase and tensin homolog (PTEN) and activation of AKT phosphorylation has also recently been shown to have some activity in increasing detection of HIV RNA in plasma shortly after dosing in a subset of patients.^65^

The main mode of action of HDACi in the treatment of cancer is the induction of apoptosis and cell-cycle arrest in rapidly dividing cells. Using the J-Lat cell line, a Jurkat cell line stably infected with HIV-1 that contains a deletion of the *Env* and *Nef* genes and encodes for expressed green fluorescent protein (EGFP), under the control of the HIV LTR^37^ we observed that following treatment with the potent HDACi, MCT1, MCT3 and oxamflatin, EGFP+ cells (i.e., cells induced to express virus) were also enriched for cells expressing activated caspase 3, annexin V and propidium iodide.^66^ However, in primary cell models HIV reactivation by vorinostat did not appear to induce death.^67^ In a recent report of elegant studies using latently infected primary T cells that overexpress BCL2, and infected with HIV-1 that contains a deletion of the *Nef* and *pol* genes and encodes for EGFP (NL4.3ΔNefΔPol-EGFP), following reactivation of HIV with vorinostat, cell did not die during 18 days of observation. Moreover, vorinostat-reactivated cells only died when co-incubated with autologous CD8 T cells from an elite controller, indicating that immune clearance is possible but requires an effective HIV-specific cytotoxic T-lymphocyte (CTL) response which is often absent in HIV-infected patients treated during chronic infection.^67^ Consistent with these findings, another study using an *in vitro* model of latently infected central memory CD4 T cells (TCM) cells, reactivation of latent virus with interleukin-2 and interleukin-7 did not cause cell death, whereas reactivation with CD3–CD28 co-stimulation did kill cells.^68^

Together, these studies indicate that viral reactivation alone may not be sufficient to induce cell death, and that other interventions may be required for TCM cells to die following viral reactivation. Other pathways that may be important for viral activation include the protein kinase C (PKC) pathway (activated by prostratin and bryostatin), the STAT5 pathway (activated by IL-7), the AKT pathway (via depletion of PTEN with disulfiram) and methylation inhibition (5-azacytadine). The effects of activation of each of these pathways and cell death need to be further explored.

### Gene Therapy Approaches

There are three general approaches that use gene therapy to attempt to cure HIV. The first is a gene knockdown to reduce the expression of a host protein that HIV requires to complete its life cycle. An example of such an approach is to use zinc finger nucleases (ZFN) to degrade the message for either CCR5^69^ or CXCR4^70^ and render these cells resistant to either R5 or X4 HIV, respectively. Several early phase trials of autologous CD4 T cells that have been modified *ex vivo* with ZFN that knockdown CCR5 have now been reported and have demonstrated that this approach was feasible in humans, was safe and well tolerated, reduced HIV RNA rebound levels during cART interruption and induced an increase in total CD4 T-cell numbers.^71^

A second approach to gene therapy for HIV involves overexpressing proteins, which limit HIV replication and/or pathogenesis, and include chimeric TRIM5*α* molecules representing human/rhesus fusions that inhibit HIV replication *in vitro*.^72^ Another approach involves overexpressing broadly neutralizing anti-HIV antibodies. The antibody b12 has been overexpressed in humanized bone marrow/liver/thymus (hu-BLT) mice resulting in durable production of human b12, and significant improvements in CD4 T-cell numbers following HIV challenge.^73, 74^

A third gene therapy approach involves expression of chimeric antigen receptors (CARs). These were first proposed more than two decades ago and the first iteration was fusion proteins consisting of the variable immunoglobulin light chain and variable heavy chain region specific for an antigen of interest, fused to the CD3*ζ* and transmembrane domains (reviewed in Sadelain *et al.*^75^). When this chimeric receptor was expressed in T cells, upon exposure to cognate antigen and ligand binding to the variable heavy and light immunoglobulin domains, CD3*ζ* signaling occurred, causing T-cell activation. Second- and third-generation CARs also include the intercellular signaling domains of CD28, 41BB, or OX40, to provide appropriate co-stimulatory signals. Recently, T cells modified with a third-generation CAR have been reported to successfully treat patients with adult acute lymphoblastic leukemia.^75, 76^ Trials of first-generation CAR-modified T cells in HIV have now been reported with >11 years follow-up and demonstrated persistence of the CAR-modified T cells and chimeric T-cell function, albeit with minimal anti-HIV effect.^77^ Ongoing attempts to maximize antiviral activity include different receptor targets, advanced generation CARs, and application of CAR technology for use in natural killer (NK) cells.

Perhaps instructed by early experience with ART, combination-based gene therapy approaches have also been suggested. Indeed, one such approach evaluated T cells transduced with a combination of a CCR5 ribosome, silencing (si)RNA to Tat/Rev and an HIV decoy RNA, transfused post-chemotherapy conditioning to HIV-infected patients with lymphoma.^78^ In this proof of concept trial, persistence of the transgene declined precipitously within the first few weeks. Other delivery backbones and other conditioning regimens are therefore being considered.^79^ These include a variety of combination approaches (Clinical Trials # NCT 01734850, NCT 01769911).

### Immune-Based Therapies

There are two broad classes of immune-based approaches that have been proposed for therapy of HIV infection—including boosting an effective immune response or reducing immune activation. The underlying premise of boosting an effective immune response is to recapitulate HIV-infected patients who can spontaneously control viral replication (elite controllers). Spurred by multi-disciplinary collaborative groups, a variety of genes and immune functions have been associated with elite control. These associations include the Δ32 allele of CCR5,^80, 81, 82^ HLA-B5701, and HLA-B27 alleles,^83^ the NK inhibitory receptors KIR3DS1 and KIR3DL1;^83, 84^ increased expression of proteins necessary for granule exocytosis-mediated cytotoxicity,^85^ such as granzyme A, granzyme B, and perforin; and higher numbers of both plasmacytoid dendritic cells and polyfunctional T cells in elite controllers compared with non-controllers or cART-suppressed patients. While it remains unknown which of these associations are required for control of HIV, the existence of these associations has spurred attempts to recapitulate the immune phenotype, to achieve immune control of HIV.

Increased immune activation is associated with morbidity and mortality from both AIDS defining and non-AIDS defining causes.^86^ Putative causes include translocation of bacterial products across the GI tract, persistent HIV and co-infection with other pathogens, such as CMV and HCV.^86^ There is a significant correlation between markers of T-cell activation (including expression of HLA-DR, CD38, and PD-1) and markers of viral persistence (including cell-associated HIV DNA and RNA) in T cells in blood and the GI tract.^87^ However, there is no association between low level plasma viremia and markers of either T-cell or innate immune activation.^88^

It is possible that targeting inflammation may reduce virus persistence or alternatively targeting virus persistence may reduce inflammation. Although multiple studies of intensification of antiretrovirals have shown no change in HIV DNA or low level plasma viremia, two studies with intensification of raltegravir have shown a reduction in immune activation.^89, 90^ In addition, both these studies demonstrated that residual virus replication persists in ∼30% of patients on cART. Several approaches that reduce inflammation are currently being evaluated, including anti-inflammatory agents, statins, chloroquine derivatives, leflunomide, pre- and pro-biotics, growth hormone, immunotoxins, and combination approaches.

Another proposed avenue for potential immune-based therapy involves the administration of recombinant cytokines, most commonly members of the IL-2 receptor *α* subunit family: IL-2, -7, -15, and -21. In the large multi-national SILCAAT and ESPRIT studies, IL-2 therapy increased CD4 T-cell number, but neither improve CD4 T-cell function nor improve health. Of relevance to the HIV cure agenda, IL-2 therapy has also been assessed as a means of decreasing HIV burden; while detectable replication competent HIV was decreased in some patients receiving cART plus IL-2 compared with cART alone,^91^ all patients had a rapid rebound in virus following treatment cessation indicating that HIV burden was not meaningfully altered by therapy.^91^

IL-7 therapy has been tested in smaller studies, and shown to increase CD4 T-cell number and function, including increasing anti-HIV-specific CD4 T-cell function.^92, 93^ IL-7 administration caused modest increases in total intracellular HIV DNA, in proportion to the increases in CD4 T-cell number, suggesting that reservoir size was increased by homeostatic proliferation,^94^ consistent with the effects of IL-7 in *in vitro* models of HIV latency.^68^ Of interest, the increases in reservoir size were associated with increased expression of the anti-apoptotic protein Bcl2,^94^ consistent with a model of apoptosis resistance favoring HIV persistence.

Both IL-15 and IL-21 enhance innate and adaptive anti-HIV responses. IL-15 augmented both NK cell and HIV- and SIV-specific CD8 T-cell function *in vitro*, suggesting a potential role for IL-15 as an immunotherapeutic to increase anti-HIV/SIV responses.^95^ IL-15, but neither IL-7- nor IL-2-treated NK cells increased expression of TRAIL, and killing of autologous CD4 T cells, and reduced the frequency of HIV containing CD4 T cells, *ex vivo*.^96^ *In vivo*, SIV-infected macaques treated with IL-15 had increased numbers of SIV-specific CD8 T cells and increased NK cell numbers with reduced numbers of SIV-infected cells in lymph nodes consistent with an antiviral effect; however, plasma viremia was increased by 2–3 logs.^97^ Therefore, any therapeutic role of IL-15 may be limited by potentially inducing viral replication.

IL-21 has been shown to enhance antiviral NK cell and CD8 T-cell immunity in animal models of non-HIV viral infections, and has been used successfully in safety trials of patients with advanced malignancy, thereby making it an attractive candidate for anti-HIV therapy.^98^ In SIV-infected macaques, IL-21 administration increased perforin and granzyme B expression in CD8, effector CD4, and NK cell subsets; however, it remains unknown whether these favorable quantitative changes will translate into improved antiviral function and elimination of latently infected cells.

## Induction of Apoptosis of Latently Infected Cells as an Approach to Cure HIV Infection

The case of the Berlin patient is instructive and it teaches many lessons that may be applicable to cure HIV infection in a more generalizable way. First, systemic myeloablative chemotherapy and radiotherapy was used, followed by stem-cell transplant, demonstrating that induction of apoptosis by interventions such as non-selective systemic chemotherapy and radiotherapy with graft *versus* host disease (GVHD) may be a key to eradicate latently infected cells, when administered with maximally suppressive cART to prevent repopulating the reservoir.^1, 2, 3, 99^ Second, some degree of toxicity to uninfected cells might be necessary so that once HIV has been eradicated, cells and tissues killed unintentionally (bystander killing) can be repopulated, including lymphoid cells such as uninfected CD4 T cells. Third, multiple treatments may be required. A central principal of cancer chemotherapy is that treatments kill a high fraction of the cancerous cells, and therefore multiple courses of cancer chemotherapy are required so that the number of cancerous cells asymptotically approaches zero. This was the case in the treatment regimen for Timothy Ray Brown, and may likely be the case with future therapies that require induction of cell death.

### Chemosensitization

**Figure 1:**
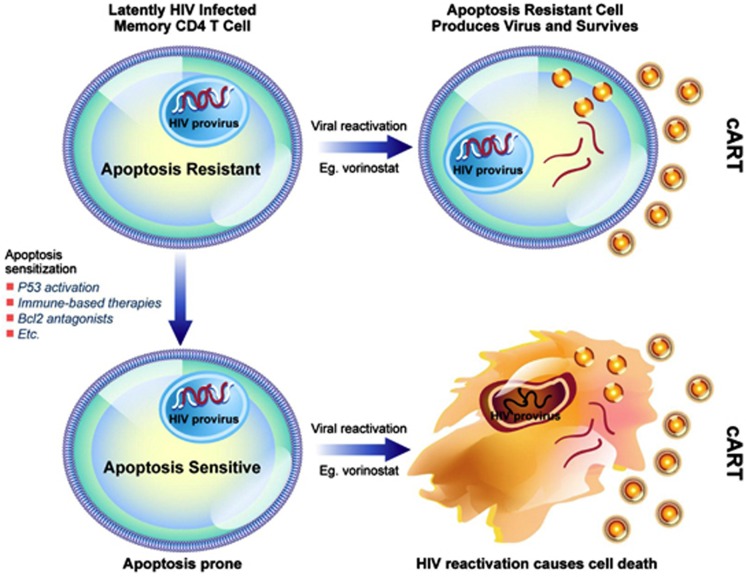
Prime, Shock, and Kill hypothesis to eradicate HIV from latently infected cells

**Figure 2:**
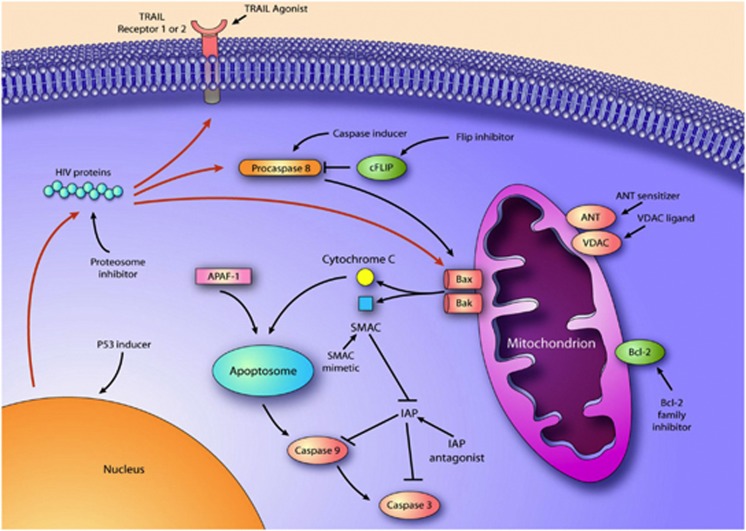
Schematic representation of the interaction of HIV proteins with different elements of the apoptosis regulatory network, and possible strategies to promote cell death following HIV reactivation

It may also be instructive to consider other similarities with cancer chemotherapy to cure HIV. Cancers that are difficult to treat are often managed with chemosensitization followed by cytotoxic chemotherapy. In the case of HIV, there is ample evidence that productive HIV replication can be cytotoxic (for example, in the case of acute T-cell infection), but also there is emerging evidence that HIV production does not always kill those cells that produce virus^100, 101^ (i.e., in the case of viral reactivation from latency). Specific reasons why remain unknown, but it is likely that some of the counter apoptotic mechanisms that occur during the development of T-cell memory or the process of chronic HIV infection are at least partially responsible. Thus, we propose to prime cells toward an apoptosis-prone phenotype, reactivate HIV pharmacologically, to induce the death of the reactivating cell—or Prime, Shock, and Kill. Possible approaches to sensitize cells to the cytotoxic effects of HIV productive replication are outlined in Figure 1. There are a multitude of chemosensitization agents used in cancer therapy, many of which are appealing to use in HIV. Some possible agents would include agents that act upon the mitochondrial permeability transition core complex (e.g., adenine nucleotide translocator (ANT) ligands, or voltage-dependent anion channel (VDAC) ligands), Bcl2 inhibitors,^102^ IAP inhibitors,^103^ proteasome inhibitors,^104^ survivin inhibitors,^105^ PI3K/AKT inhibitors,^106^ and TRAIL along with TRAIL sensitizers^107^ (Figure 2). Also, agents known to enhance the susceptibility of immune cells to apoptosis induction such as Toll-like receptor (TLR) agonists, co-stimulatory agents (e.g., anti-CD28), and agents that induce p53 warrant consideration.

### Caspase 8

One goal for the HIV cure initiative is therefore to design strategies that cause viral reactivation that then results in death of the cells which reactivate virus. We have been studying the involvement of Caspase 8 in the induction of cell death during productive HIV infection, and have observed that during acute infection of CD4 T cells, HIV protease cleaves Caspase 8^108^ to create a unique protein fragment, Casp8p41. Casp8p41 translocates to mitochondria and independently induces mitochondrial permeability leading to apoptosis, as well as to NF*κ*B activation.^53^

If latently infected CD4 T cells that reactivate HIV do not die, then it is possible that the Casp8p41 pathway of death is not operational in such cells. Insight into why this pathway is not activated following viral reactivation may lie in observations that short-term activation of CD4 T cells with antigen results in upregulation of procaspase 8 whereas long-term activated cells that acquire a memory phenotype downregulate procaspase 8, and become apoptosis resistant.^109^ Thus, the low levels of procaspase 8 and intrinsic apoptosis resistance of memory CD4 T cells might explain why such cells do not die following HIV reactivation. Developing strategies to increase Caspase 8 expression in cells that harbor latent HIV and then inducing viral reactivation with agents such as HDACi should lead to testable ‘sensitizing' strategies designed to enhance death of latently infected cells that are induced to reactivate virus.

### TLR stimulation

The rationale for this approach is that TLR-ligand stimulation of T cells will lead to NF*κ*B activation and cytokine production,^110^ which may upregulate pro-apoptotic regulatory proteins and downregulate anti-apoptosis regulatory proteins, and render TCM cells sensitive to the cytotoxic effects of productive HIV replication. This model is particularly appealing, given recent data that mRNA for TLR1, TLR2, TLR3, TLR4, TLR5, TLR7, and TLR9 has been identified in primary CD4 T cells, and stimulation of resting CD4 T cells with agonists of TLR2, TLR4, and TLR5 triggers interferon-*γ* production.^111^ Also, TLR5 stimulation of TCM cells isolated from HIV-infected individuals leads to HIV reactivation, although the viability of these cells after stimulation with the TLR5 ligand flagellin was not specifically examined.^112^ Likewise, as long ago as 1987, double-stranded RNA (a TLR3 ligand) was shown to have significant anti-HIV activity and caused >90% of productively infected cells to die because of HIV-related cytopathic effects while having no effect on the viability of uninfected cells.^113^

### Auranofin

Another intriguing possibility concerns auranofin, a gold-based compound successfully used for years to treat rheumatoid arthritis. Although the mechanism of action of auranofin is incompletely understood, some reports link it to altered regulation of p53 pathways.^114^ Therefore, one might predict that auranofin could alter the cellular millieu to favoring apoptosis. Auranofin, in addition to ART (tenofovir, emtricitabine, and raltegravir), in an SIVmac251-infected macaque model induced activation and death of resting memory cells and reduced the amount of cell-associated SIVmac251 DNA in auranofin-treated monkeys compared with those who received ART alone.^115^ Despite these promising findings, the mechanism by which these latently infected cells were dying was not investigated.

## Conclusion

HIV is a disease characterized by altered cell death, wherein the majority of CD4 T cells and other cell types die at an accelerated rate, leading to a significant immune dysregulation. On the other hand, HIV infection fails to cause the death of all of the cells that it infects. This allows for the development of long-lived latently infected HIV reservoirs and the long-term persistence of HIV in the presence of ART. Enhanced understanding of the molecular mechanisms that allow HIV to survive in latently infected cells will allow for interventions that are designed to reverse virus persistence. These interventions could effectively reactivate latent HIV and simultaneously induce cell death—ultimately leading to reduction in the number of latently infected cells and potentially a cure for HIV infection.

## Figures and Tables

**Table 1:** Approaches to HIV cure

**Approaches to HIV cure**	
Gene therapy	Knockdown of proteins required for HIV replication. For example, CCR5
	Overexpression of restriction factors. For example, Human: Rhesus chimeric TRIM5a
	Engineered T-cell receptors. For example, Third-generation chimeric antigen receptors
Immune based	Therapeutic vaccination
	Cytokine therapy. For example, 1h7, IL15
	Anti-inflammatory agents
	Growth hormone
HIV reactivation	HDAC inhibitors—For example, SAHA
	TLR agonists
	PKC activation
Cytotoxic approaches	Autologous stem-cell transplant
	Allogeneic stem-cell transplant

**Table 2:** Compounds that activate latent infection

**Mechanism of action**	**Name**	**Clinical trials**^a^	**Reference**
Histone deacetylase inhibition (HDACi)	Valproic acid		^43, 48, 66, 116, 117^
	Trichostatin A		^43, 116, 117^
	Vorinostat	2	^43, 48, 63, 116, 117, 118, 119^
	Sodium butyrate		^116, 117^
	Oxamflatin		^66, 116^
	MCT-1 and 3		^66^
	MRK1, 10, 11, 12, 13, 14		^120^
	MC compounds		^118^
	Givinostat		^121^
	Givinostat analogs		^121^
	Scriptaid		^116, 117^
	NCF-51		^122^
	Belinostat		^121, 123^
	Panabinostat	1	^123, 124^
	Entinostat		^117, 118, 124^
	Apicidin		^116, 117^
	CG05, CG06		^119^
	Droxinostat		^116^
	M344 Romedepsin	In devpt	^125^
Methylation inhibitors	5-aza-2'deoxycytidine (Aza-CdR)		^126^
	BIX-01294		^127^
	Chaetocin		^127, 128^
			^129^
NF*κ*B activators	Prostratin		^117, 125, 127, 130^
	TNF*α*		^130^
Protein kinase C modulators	Bryostatin		^131^
Akt/HEXIM-1 modulators	Hexamethylbisacetamide (HMBA)		^132, 133^
	Disulfiram	1	^134^
BET bromodomain inhibitors	JQ1		^135^
			
Immune modulation	IL-7	6	^130, 136^
	IL-15		^137^
	Anti-PD1 Anti-PDL1	In devpt In devpt	^138^
Combinations	AV6+Valproic acid		^139^
	Bryostatin+Valproic acid		^131^
	HDACi+Prostratin		^117, 125, 130^
	Prostratin+IL-7		

All compounds have demonstrated activity *in vitro* in either latently infected cells lines, latently infected primary T cells, and/or resting CD4+ T cells from HIV-infected patients on cART.

a Completed or currently active trials in HIV-infected patients on cART (source clinicaltrials.gov).

## Abbreviations

HIV: human immunodeficiency virus
ART: antiretroviral therapy
EBV: Epstein Barr Virus
CMV: cytomegalovirus
HSV: herpes simplex virus
HPV: human papilloma virus
BMT: bone marrow transplant
NS: nonstructural
cFLIP: cellular FLICE inhibitory protein
XAIP: x-linked apoptosis inhibitory protein
TRAIL: TNF-related apoptosis inhibitory protein
TNF: tumor necrosis factor
GI: gastrointestinal
CNS: central nervous system
IUPM: infectious units per million
FADD: Fas-associated death domain
TCR: T-cell receptor
MHC: major histocompatibility complex
AICD: activation-induced cell death
TEM: effector memory T cell
TCM: central memory T cell
SDF-1*α*: stromal-derived factor-1*α*
HDACi: histone deacetylase inhibitor
PTEN: phosphatase and tensin homolog
CTL: cytotoxic T-lymphocyte
EGFP: expressed green fluorescent protein
ZFN: zinc finger nucleases
hu-BLT: humanized bone marrow/liver/thymus mouse
NK: natural killer
ANT: adenine nucleotide translocator
VDAC: voltage-dependent anion channel
TLR: Toll-like receptor
cART: combination antiretroviral therapy
